# The Role of Physical Activity on Parental Rejection and Body Image Perceptions

**DOI:** 10.3390/ijerph17072176

**Published:** 2020-03-25

**Authors:** Celia K. Naivar Sen, Duygu Gurleyik, Elia Psouni

**Affiliations:** 1 Department of Psychology, Ozyegin University, Istanbul 34794, Turkey; celia.sen@ozyegin.edu.tr (C.K.N.S.); duygu.gurleyik@ozyegin.edu.tr (D.G.); 2 Department of Psychology, Lund University, Lund SE-22100, Sweden

**Keywords:** parental rejection, physical activity, body image, social physique anxiety, adolescents

## Abstract

The present study investigated the potential moderating role of physical activity on the relationship between parental rejection and poor body image perceptions. Late adolescents and young adults from Turkey (*N* = 373; 256 females/117 males) reported their memories of upbringing (Egna Minnen Beträffande Uppfostran/EMBU) related to both their mother and father, respectively, levels of physical activity (International Physical Activity Questionnaires/IPAQ), and body image perception (Body Cathexis Scale/body dissatisfaction and Social Physique Anxiety Scale/SPAS). EMBU mother and father rejection scores were combined and dichotomized, placing participants into high and low rejection groups. Multiple analysis of covariance, controlling for gender and body mass index, showed that high parental rejection was associated with poorer overall body image perception (*η^2^* = 0.09; *η^2^_Body Dissatisfaction_* = 0.09; *η^2^_SPAS_* = 0.04), whereas higher physical activity was linked to better body image perception *η^2^* = 0.02; *η^2^_Body Dissatisfaction_* = 0.04; *η^2^_SPAS_* = 0.03). While level of physical activity did not mediate the negative relationship between parental rejection on body image perceptions, very physically active individuals recalling high parental rejection displayed body image perceptions similar to participants with low parental rejection. Thus, although higher parental rejection is related to poorer body image perception, interventions targeting regular physical activity may help buffer against these negative effects.

## 1. Introduction

Body image is a complex and dynamic construct of one’s perception of their own body that can be either positive or negative [1]. Body image is one aspect of self-perception that appears to be strongly influenced by parental input [2] with negative body image perceptions established in children as early as age six [3], peaking in adolescence, and persisting into adulthood [4,5]. An estimated 20% to 46% of adolescent girls and young adult women and 12% to 26% of adolescent boys and young men experience negative body image perceptions [4,6,7,8,9]. Poor body image predicts physical and mental health problems including frequent dieting [10], eating disorders [11], weight gain [12], low self-esteem [13], depression [14], and suicidal ideation [15].

Given the prevalence and early onset of negative body image, it is crucial to understand its developmental origins and also to identify factors that may help moderate negative body image perceptions. One potential factor associated with moderation in negative body image perception is physical activity (PA) [16]. Thus, the current study investigates the developmental aspect of negative body image via parental rejection in late adolescence and young adulthood while also examining the potential buffering effect of physical activity.

### 1.1. Body Image Perception: The Role of Parents

Body image encompasses a person’s own body-related attitudes and perceptions including thoughts, feelings, and behaviors [17]. Body image perception lies on a continuum ranging from healthy body perceptions marked by reasonably accurate and mostly positive self-evaluations to unhealthy body image marked by inaccurate and mainly negative appraisals [18]. Although weight and body mass index (BMI) may be components of body image [19], research indicates that the psychological perception of one’s body is more important than one’s physical dimensions, with body image perceptions hinging on a person’s subjective evaluation of what it means to have their particular kind of body within their culture [2,20]. Thus, beyond weight and BMI, several psychological conceptualizations of body image perception exist in the literature including: body dissatisfaction, weight satisfaction, size perception accuracy, appearance satisfaction, body satisfaction, appearance evaluation, appearance orientation, body concern, drive for thinness, body esteem, body schema, drive for muscularity, and social physique anxiety [21,22,23,24]. As the public health implications of negative body image perceptions accumulate, research is being directed toward understanding the formative factors involved in the development and maintenance of negative body image perception [25].

Body image perception is a dynamic characteristic changing over the lifespan, grounded in childhood experiences and critically developing during adolescence and early adulthood [26,27,28]. As a component of the individual’s self-representation, body image perception is influenced by the way others perceive and reflect our image back to us [29,30,31], starting with the individual’s interactions with primary caregivers during early childhood. Attachment theory [32,33] provides a theoretical framework for understanding the development of self-representations within the context of early parent–child relationships [34]. As mental models of early relationships, attachment representations are thought to also include self-perceptions of one’s value and worthiness, giving rise to expectations that influence beliefs and feelings about the self, with different experiences with caregivers involving different mental models of self. For instance, when primary caregivers do not answer, deny, minimize or selectively dismiss the child’s needs, the child may develop a mental schema in which they are not worthy of sensitive care and love [35,36]. Consistently, research has shown that the secure attachment that develops in the absence of such dismissing parental behaviors is predictive of positive feelings and beliefs about the self and positive self-perceptions [34,37,38,39,40,41,42].

Research concerning parental influences on body image perception is, however, curiously scarce, as most studies have focused on body image and weight specific communication including teasing or negative body image modeling by parents [43,44], overlooking potential influences on the child’s self-perception through more overarching parental behaviors. It is therefore unknown to what extent parental dismissal, for instance, through minimization, underestimation, or ridiculing of the child and their affective states and needs, may contribute to negative body image perceptions.

As children reach adolescence, core self-perceptions are combined with internalized social body ideals as they are portrayed by not only parents, but also by friends and the media [45]. To this aim, the tripartite influence model [22] suggests that three primary factors together influence body image perceptions: peers, media, and parents. Indeed, empirical evidence supports the roles of peer and media involvement in the construction and maintenance of negative body image perceptions in late adolescents and young adults [46]. The commonly resulting body dissatisfaction, defined as a person’s negative thoughts and feelings about their body [47], is frequent in many cultures, beginning as early as the age of eight. At the core of body dissatisfaction is a discrepancy between one’s current perceived body image and one’s ideal body; and it is suggested that this discrepancy is stronger and more frequent among women due to cultural pressures to conform to idealized body shapes as well as frequent exposure to idealized body images through the media [48,49]. Body dissatisfaction is often associated with attempts to alter body shape and weight, but also with attempts to hide one’s body shape, leading many individuals to avoid activities that would involve exposing their bodies [20]. The psychological principle behind such behavioral tendency is a worry that others may negatively evaluate one’s own body [24,50], which is captured in the concept of social physique anxiety (SPA) [51]. In that sense, SPA captures the influence of peers/other people. Thus, SPA influences individual behaviors when other people are present to evaluate physical appearance. SPA is linked to poor physical self-esteem [52], negative body image [53], dissatisfaction with appearance and weight [54], negative attitudes toward eating [55], and disordered eating [56].

### 1.2. Physical Activity on Body Image

While parental rejection may negatively influence body image perception, PA may have a positive role. Regular PA has been consistently associated with better physical and mental health [57,58]. For example, research shows that individuals who engage in recommended levels of PA on a regular basis had positive mood states [59], enhanced health-related quality of life [58], better weight management, and increased physical self-esteem compared to their non-active counterparts [16]. The standard guideline of 150 minutes of moderate PA (e.g., brisk walking, jogging) per week is sufficient to receive these beneficial health outcomes. Additionally, there is ample evidence of the importance of body image perception and other performance-related aspects of physical self-perception for PA participation in populations of different ages [60,61] and in adolescents and young adults specifically (e.g., [62]).

While the physical health benefits of PA are well documented, the relationship between PA participation and body image perception is more complex [63]. When discussing the potential impact of PA on body image perception, research often gravitates toward aesthetics, improved appearance, and achieving cultural ideals of body form [20]. However, in addition to changing one’s physical appearance, PA may change the way people feel about their bodies, as it is associated with positive health benefits and increased physical capabilities (e.g., vigor, energy). Indeed, research supports a link between PA and positive body image (e.g., [64,65,66,67,68]) such that adolescents regularly engaging in PA are less likely to suffer from poor body image. On the other hand, a recent review [65] pointed out that while PA may positively influence body image perceptions of people who are dissatisfied with their body and wish to lose weight, get toned, and have a healthy appearance, but in other cases, PA may instead aggravate negative body image perceptions, especially for those not attaining their ideal body or those high in SPA. Individuals with high level SPA may, for instance, shy away from environments where their body can be gauged by others. Thus, when investigating the relationship between body image and PA, aside from body dissatisfaction, taking into account SPA might be crucial. Therefore, the current study looks to clarify the role of PA and to investigate if regular PA might moderate the negative consequences of parental rejection on the body image of late adolescents and young adults including both body dissatisfaction and SPA.

### 1.3. Current Study

In the current study, we ascertained late adolescents’ and young adults’ recalled levels of parental rejection, current PA, and body image perception as measured by body dissatisfaction and SPA, in order to understand how parental rejection and PA combine to influence the perception of body image. Based on the tripartite model, we expected an influence from parents. Unlike other studies that have focused on parental influences such as teasing or criticisms about weight and appearance [43,44], we focused here on the role of the entire repertoire of parental behaviors that constitute rejection, and expected participants who recalled several experiences of parental rejection to also hold more negative body image, compared to participants with limited experiences of parental rejection. Acknowledging that negative body image can contribute to lower levels of PA, we also recognize that PA is a behavior of choice that can be modified throughout the lifespan. Thus, we further hypothesized that higher levels of current PA may buffer the expected negative impact of past parental rejection on current body image when controlling for gender and BMI, given that moderate-to-high levels of PA may contribute to changing current individual perceptions and feelings about the body, thanks to positive health benefits and increased experienced vigor and energy. Based on previous evidence that the role of PA is uncertain in individuals with high SPA, after testing for moderation, we ruled out the possibility of PA having a mediating role.

## 2. Materials and Methods

### 2.1. Participants

Participants were undergraduate students *(N* = 373; 256 females/117 males) recruited from a private four-year university in Istanbul, Turkey. The participants’ average age was 21.31 years (*SD* = 1.76, range 18–27 years). As the study was conducted in Turkish, only individuals fluent in Turkish participated.

### 2.2. Materials

#### 2.2.1. Demographic Information

Participants reported their age, gender, and height and weight for BMI calculation [69].

#### 2.2.2. Short-EMBU (Egna Minnen Bettrafende Uppfostran, My Memories of Upbringing)

The Short-EMBU (Egna Minnen Bettrafende Uppfostran) [70] was used to measure the participants’ current memories of their parents’ child-rearing behaviors during childhood). It consists of 23 items reflecting three subscales: rejection, overprotection, and emotional warmth. Items use a 4-point Likert type scale ranging from 1 (never) to 4 (most of the time). Only the seven-item parental rejection subscale was considered in the current study, administered to assess maternal and paternal rejection, respectively (e.g., “My mother/father treats me in such a way that I feel ashamed”). In a multinational study, the Short-EMBU displayed good reliability and validity with maternal and paternal rejection reliability scores ranging from 0.74 to 0.79 and 0.72 to 0.77, respectively [70]. The scale, adapted into Turkish, had Cronbach’s alphas of 0.64 and 0.73 for maternal and paternal rejection, respectively [71]. In the current study, Cronbach’s alpha for both scales was 0.83.

#### 2.2.3. International Physical Activity Questionnaire-Short Form (IPAQ-SF)

The IPAQ-SF, a 7-item self-reporting survey, measures durations and frequencies of sitting, walking, moderate, and vigorous intensity activities during the past seven consecutive days (e.g., brisk walking, cycling, stair climbing, etc.). A total score is then obtained by multiplying time (in minutes) engaging in the activity with metabolic equivalent of task (MET) values of 3.3, 4, and 8 for walking, moderate PA, and vigorous PA, respectively. Based on the obtained continuous score, participants can also be categorized as being highly (≥3000 MET-min/wk), moderately (≥600 MET-min/wk) or low (<600 MET-min/wk) in activity. The recommended guideline of 150 minutes of moderate PA per week (e.g., 30 minutes, 5 days) is approximately equivalent to 600 METS or the moderate category of PA. Test-retest reliabilities range from 0.71 to 0.89 for the English version [72] and 0.69 for the Turkish translation [73].

#### 2.2.4. Body Cathexis Scale (BCS) for Body Dissatisfaction

The 40-item body cathexis scale (BCS) [21] was used to rate the participants’ satisfaction with various body parts and aspects of their body such as “face”, “thighs”, and “weight” on a 5-point Likert scale (“1” = “very satisfied” and “5” = “very dissatisfied”). For score calculation, all items were summed with a theoretical range from 40 to 200. Cronbach’s alphas ranged from 0.78 to 0.87 with a test–retest reliability of 0.89 for the English version [74] and 0.91 for the Turkish translation [75]. Cronbach’s alpha for the current study was 0.93.

#### 2.2.5. Social Physique Anxiety Scale (SPAS)

The 12-item SPAS [76] was developed to assess the degree to which people experience anxiety when others observe or evaluate their physiques. Items like “in the presence of others, I feel apprehensive about my physique or figure” were rated on 5-point Likert scales from 1 (not at all characteristic of me) to 5 (extremely characteristic of me). SPAS demonstrated both high internal and test–retest reliability of 0.90 and 0.82, respectively [24]. The Turkish adaptation demonstrated high internal and test–retest reliability of 0.80 and 0.88, respectively [77]. Cronbach’s alpha for the current study was 0.91.

### 2.3. Procedure

The study was announced via email and data were collected using Qualtrics in a proctored computer lab. Written ethical approval was granted by the Ozyegin University’s Research Ethics Committee (2019/4). Participants were informed verbally and in writing about the nature and aims of the study, and that their participation was anonymous and voluntary. They were reminded that they could terminate participation at any time without any explanation required. For participation, participants received course credit in a class of their choice. All collected data were stored on password protected units and treated confidentially. Only the authors had access to the data. The survey took approximately 20 min to complete.

### 2.4. Data Preparation and Analysis

For data analysis, we used IBM SPSS 20 (IBM, Armonk, NY, USA) [78] and Jamovi (Jamovi, https://www.jamovi.org) [79]. As small (score) differences in the amount of recalled parental rejection may not result in robust differences in quality of growing environment, the variable parental rejection was dichotomized. Thus, the fourteen rejection items (for memories regarding mother and memories regarding father) were averaged and the median split (*Median* = 1.29, *M* = 1.39, *SD* = 0.40) was used to recode each individual as high or low on parental rejection. For hypothesis testing, we tested the direct effect of recalled parental rejection (high versus low), and the role of PA (low, moderate, and high) on body image outcomes (body dissatisfaction and SPA). Moderation was carried out using MANCOVA with subsequent graphs via SPSS (IBM, Armonk, NY, USA), and mediation was carried out via Jamovi.

## 3. Results

### 3.1. Descriptive Statistics

Participants reported on average low to moderate levels of parental rejection (*M* = 1.39; *SD* = 0.40). The group with low rejection reported an average score of 1.11 (*SD* = 0.08), while the group with high rejection reported 1.65 (*SD* = 0.41). The two groups were significantly different in terms of recalled rejection (*t*_(371)_ = 17.89, *p* ≤ 0.001). Participants had on average low to optimal BMI, falling within the 18.5 to 24.9 healthy weight range. Both body dissatisfaction (*M* = 96.94; *SD* = 23.10) and SPA (*M* = 32.99; *SD* = 10.30) were above average, and the sample was active with only 17.2% not meeting minimum activity guidelines, while 41.3% reported moderate and 41.6% reported high levels of weekly PA. Body dissatisfaction and SPA were strongly correlated (*r* = 0.66). As expected, there was a gender difference for both body dissatisfaction (*t*_(371)_ = 5.00, *p* ≤ 0.001) and SPA (*t*_(371)_ = 2.71, *p* ≤ 0.01), with females reporting higher levels on both constructs (*M_Body Dissatisfaction_* = 34.73, *SD* = 10.21; *M_SPA_* = 99.11, *SD* = 22.61) compared to males (*M_Body Dissatisfaction_* = 29.16, *SD* = 9.44; *M_SPA_* = 92.19, *SD* = 23.53).

### 3.2. Moderation Analysis: Multiple Analysis of Covariance (MANCOVA) and Graphical Representations

#### 3.2.1. MANCOVA

The potential moderating effect of PA on the link between recalled parental rejection and negative body image was investigated via a 2 × 3 MANCOVA. Parental rejection (high vs. low) and PA (low vs. moderate vs. high) were grouping variables, while negative body image, comprised of body dissatisfaction and SPA, were the dependent variables. Due to the gender differences in body image perceptions and the likely correlation of BMI with negative body image, these two constructs were controlled for in the analysis.

The multivariate link between recalled parental rejection and negative body image was significant (Table 1) with significant effects on both body dissatisfaction (*M_low_* = 90.68, *M_high_* = 104.92) and SPA (*M_low_* = 31.26, *M_high_* = 35.19). Therefore, the first hypothesis regarding participants with higher parental rejection displaying higher levels of negative body image perceptions was supported. Furthermore, the multivariate effects of PA on negative body image were significant, as were the univariate effects of PA on body dissatisfaction (*M_low_* = 102.22, *M_moderate_* = 99.68, *M_high_* = 91.50) and SPA (*M_low_* = 34.26, *M_moderate_* = 34.22, *M_high_* = 31.20). Post-hoc analysis (Bonferroni) indicated that the differences between the low and average PA groups were not significant, but the high PA group differed significantly from the other two. Hence, our second hypothesis, stating that participants with higher levels of PA would display lower levels of negative body image perceptions, was partly supported. Contrary to our hypothesis, the effect of the interaction between parental rejection and PA was not significant.

#### 3.2.2. Graphical Representations

**Figure 1:**
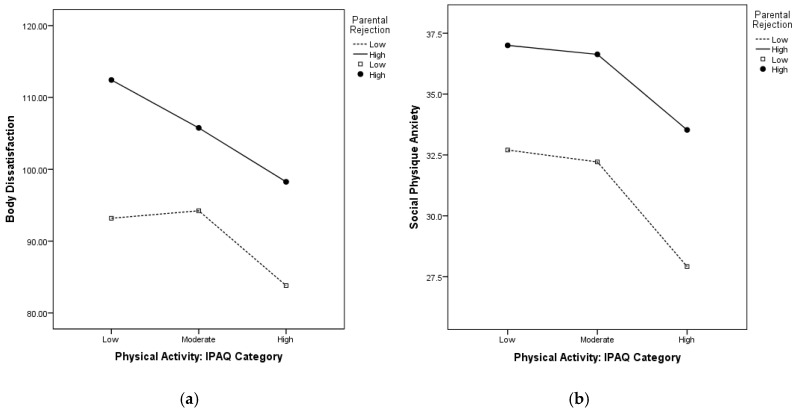
The relationships between parental rejection and physical activity on (**a**) body dissatisfaction and (**b**) social physique anxiety (*N* = 373).

Graphical representation of the interaction between recalled rejection and PA on body dissatisfaction and SPA helped clarify the role of PA on this relationship. For both body dissatisfaction (Figure 1a) and SPA (Figure 1b), the group of participants who recalled higher parental rejection displayed higher levels of poor body image. For this group (full line), any increase in PA level was associated with less body dissatisfaction. Body dissatisfaction scores of those high in rejection and high in PA levels were close to those of their lower rejection and low/moderate PA peers. Similar effects were seen for SPA; however here, only participants who reported high levels of parental rejection, who also reported high levels of PA had a drop in SPA. These trends reveal a positive influence of PA.

### 3.3. Mediation Analysis

To rule out possible mediation of PA between parental rejection and negative body image perceptions, mediation analysis was carried out. For body dissatisfaction analysis, the direct effect of parental rejection on body dissatisfaction was significant, as was the relationship between PA and body dissatisfaction (Table 2). While the relationship between rejection and PA was marginally significant, the indirect effect and total effects were not significant, indicating that PA did not mediate the link between parental rejection and body dissatisfaction.

For SPA, the direct effect of parental rejection on SPA was significant, as was the relationship between PA and SPA (Table 3). However, the indirect effect was not significant, indicating that PA did not mediate the link between parental rejection and SPA.

## 4. Discussion

The present study aimed to define the moderating role of PA in the relationship between parental rejection and negative body image in late adolescents and young adults. As hypothesized, parental rejection was linked to more negative body image perceptions, encompassing both body dissatisfaction and social physique anxiety. Higher PA levels were linked to lower levels of negative body image perceptions, but as predicted, they did not mediate the link between recalled parental rejection and negative body image. However, the positive role of PA was demonstrated in that participants recalling higher parental rejection, who also were very physically active, displayed body image perceptions similar to participants who recalled little or no parental rejection.

### 4.1. Parental Rejection and Body Image Perceptions

Individuals who recalled and reported more frequent parental rejection during childhood reported significantly more body dissatisfaction and higher levels of SPA, in line with the tripartite influence model [22]. These findings expand previous evidence of the negative effects of parental communication specifically focusing on body and weight including teasing, or negative body image modeling by parents [43,44] on body image. While research has demonstrated the role of parental rejection in the development of disordered eating [80], only one other study could be found showing a link between maternal rejection on body dysmorphic disorder, a clinical disorder characterized by a preoccupation with a defect in one’s appearance [81]. Our findings suggest that the link between parental behaviors and the child’s internalized self-representations may not be domain-specific. Even in the absence of parental comments directly derogating the child’s body form or teasing about the child’s weight, parenting practices that are insensitive of the child’s emotional states, needs, or perspectives, thus communicating rejection of the child, contribute to more negative body image perceptions.

Adjusting to consistent experiences of rejection, individuals growing up in rejecting parental environments become dismissive. As no one seems to care about these individuals’ negative feelings or makes an attempt to mitigate them (soothing), they become conditioned to care less about their own (and others’) feelings as well as learn to care less about other people and their inputs [34,82,83]. In the present study, however, the group who recalled higher parental rejection did not report high levels of rejection. It is thus plausible that these individuals experienced as children a fair amount of rejection but also, at other times, kindness and sensitivity. Due to this inconsistency, it is, according to attachment theory, not possible for the child to count on the parent’s sensitive care and acceptance, resulting in behaviors aimed at keeping the parent engaged [32,33], for instance, through exaggerated expressions of negative affect and needs, and a preoccupation with gaining and maintaining the parents’ and others’ approval [63,82]. The strong effect of recalled parental rejection on social physique anxiety supports this.

### 4.2. The Role of Physical Activity

Parental rejection was moderately linked to lower PA levels. This is in line with meta-analytical evidence that parental influences such as the encouragement of PA, instrumental assistance (i.e., help with transportation or providing equipment access), and modeling (i.e., observational learning via parents of PA or sedentary behaviors) on the children’s PA are present but weak [84]. In fact, among those parental behaviors, parental encouragement of PA appears to have the strongest link to increased levels of PA [84]. Our results further these findings by suggesting that it is not only PA-specific encouragement that positively affects children’s PA levels, but also general encouragement, here denoted as the absence of rejection and criticism. As the present study did not include specific measures of the parents’ PA levels and related recreational activities, it was not possible to evaluate the potential role of sharing such activities on the children’s later PA levels.

Importantly, consistent with our hypothesis, our results suggest that PA may have a moderating role on the negative impact of parental rejection on body image. Participants with higher levels of recalled parental rejection benefit from PA, especially from high PA levels, and even those with moderate levels of PA seemed to benefit in terms of lower body dissatisfaction. Notably, very physically active individuals recalling high parental rejection displayed body image perceptions similar to participants with low parental rejection. Thus, while confirming the link between high levels of parental rejection during childhood and negative physical self-representation, and despite findings that negative body image may contribute to lower PA, our results also highlight that behavior modification through interventions targeting regular PA may help moderate the negative effects of parental rejection on individual self-representations.

PA was negatively associated with body dissatisfaction and greater levels of SPA, supporting other findings that engaging in PA may alleviate negative body image perceptions [64,65,66,67,68]. Our findings further revealed that, independent of the amount of recalled parental rejection, only participants with high PA levels had substantially lower SPA levels. For both low and moderately active groups, SPA remained relatively stable, especially for individuals reporting high parental rejection. Given other evidence of a complicated relationship between PA and SPA [65], this finding is not surprising. As SPA increases with the presence of others and their subsequent potential judgement, individuals with high levels of SPA may shy away from environments where their body can be judged [85].

While high levels of PA may help reduce SPA, motivating individuals with high SPA requires sensitivity and respect for the anxiety reducing techniques used by such individuals such as wearing loose clothing and exercising in private, during off-peak gym hours [86], or in more comfortable environments (e.g., only women gyms). Shifting focus away from appearance benefits, and focusing instead on health benefits has been shown to be particularly helpful to this end [87,88]. Thus, interventions targeting PA increase must be sensitive to these strategies and behavioral patterns.

### 4.3. Limitations

Due to the cross-sectional nature of our design, no claims of causality can be made based on the current study findings. While findings suggest a buffering effect of PA so that late adolescents who recalled high levels of parental rejection, but who were very physically active, reported body image perceptions similar to participants who recalled little or no parental rejection, longitudinal intervention studies with parent/child observations would be necessary in order to address causality. Additionally, the study sample was unbalanced in terms of gender, as male participants represented only 31.4%. A balanced sample in which also non-binary late adolescents would be represented would increase generalizability.

Furthermore, data collection was based on self-reporting, which makes it impossible to exclude individual bias. For instance, although the IPAQ is a widely used measure for assessing levels of physical activity with well-established standards and evidence of reliability and validity, it is also known via systemic review that participants are prone to incorrectly estimate their PA levels [89]. While underestimates are less common, at most 28% underestimation, individuals tend to overestimate their activity at a rate up to 106% of their reported activity (range of 36% to 173%) [89]. Thus, research indicates that up to half of participants claiming moderate PA levels may actually not be physically active enough to meet the requirements for this classification, while up to one-third of those reporting vigorous activity may not be accurately classified [90]. In the current study, there was little difference between the low and moderate PA groups. Perhaps this lack of difference could have resulted from individuals with low levels of PA having over-reported and been incorrectly classified as moderately active. More sensitive measures like computerized or manual activity logs may thus be necessary as complements to IPAQ [91] to assist in more accurate classification and improved results.

Importantly, recalled parental behaviors, as generated by a questionnaire such as the EMBU, may be an indicator but not an accurate measure of the amount of rejection experienced during childhood. First, there are large individual differences in the extent and detail of autobiographic memory [92]. Second, there are well-documented, systematic differences in terms of what types of memories individuals rehearse, and therefore reinforce, during adolescence [63]. Finally, circumstantial influences such as current positive or negative experiences—and related affect—in relation to the parents, may have influenced the participants’ responses in the moment. Other resource-efficient approaches to assessing the quality of the participants’ experiences with their parents during childhood [82] may be more suitable to integrate in future investigations.

## 5. Conclusions

The present study revealed that late adolescents and young adults who recalled more general parental rejection during childhood, displayed more negative body image perceptions and social physique anxiety. As high levels of PA were linked to lower levels of negative body image perceptions in participants who recalled higher parental rejection, future interventions may look to increase PA, particularly among children growing up in inconsistent and rejecting environments. As body image perceptions are formed in early childhood but become less flexible after late adolescence, intervention prior to late adolescence is essential. For instance, through participation in team-sports and the positive experiences of coaching and peer support, body image dissatisfaction may diminish [93]. This may be particularly important for girls; whose body image perceptions are more negative.

Finally, as the present findings indicate that non-body-specific inattention to, or dismissal of, the child’s emotions, contributes to negative body image perceptions, early interventions targeting PA may not only moderate these specific negative influences regarding body image and physical self-representations, but also help mitigate the negative impact of parental rejection on other facets of the individual’s self-representation and self-image.

## Figures and Tables

**Table 1:** Multiple analysis of covariance table for negative body image (body dissatisfaction and social physique anxiety), *N* = 373.

Effect	*F*	*P*	*η*2
Parental Rejection	17.45	0.0001	0.09
Rejection on Body Dissatisfaction	34.92	0.0001	0.09
Rejection on SPA	14.65	0.0001	0.04
PA	4.35	0.002	0.02
PA on Body Dissatisfaction	8.19	0.0001	0.04
PA on SPA	5.05	0.007	0.03
Parental Rejection x PA	0.92	0.451	0.01
Parental Rejection x PA on Body Dissatisfaction	1.04	0.354	0.01
Parental Rejection x PA on SPA	0.626	0.535	0.003
Covariates			
Gender	28.68	0.0001	0.14
Gender on Body Dissatisfaction	9.18	0.003	0.03
Gender on SPA	54.16	0.0001	0.13
BMI	29.49	0.001	0.14
BMI on Body Dissatisfaction	7.83	0.005	0.02
BMI on SPA	54.54	0.0001	0.13

**Table 2:** Indirect and total effects: parental rejection ⇒ IPAQ ⇒ body dissatisfaction (*N* = 373).

Type	Effect	Estimate	*SE*	95% C.I.		*Β*	*z*	*p*
				Lower	Upper			
Indirect	Rejection ⇒ PA ⇒ Dissatisfaction	0.48	0.27	−0.05	1.02	0.02	1.78	0.075
Component	Rejection ⇒ PA	−0.07	0.04	−0.15	0.00	−0.10	−1.95	0.051
	PA ⇒ Body Dissatisfaction	−6.62	1.52	−9.61	−3.64	−0.21	−4.35	<0.001
Direct	Rejection ⇒ Body Dissatisfaction	7.06	1.11	4.89	9.23	0.31	6.38	<0.001
Total	Rejection ⇒ Body Dissatisfaction	0.48	0.27	–0.05	1.02	0.02	1.78	0.075

Note. Confidence intervals computed with the method: Standard (Delta method).

**Table 3:** Indirect and total effects: parental rejection ⇒ PA ⇒ SPA (*N* = 373).

Type	Effect	Estimate	*SE*	95% C.I.		*Β*	*z*	*p*
				Lower	Upper			
Indirect	Rejection ⇒ PA ⇒ SPA	0.17	0.10	−0.03	0.37	0.02	1.69	0.091
Component	Rejection ⇒ PA	−0.07	0.04	−0.15	0.00	−0.10	−1.95	0.051
	PA ⇒ SPA	−2.37	0.70	−3.75	−1.00	−0.17	−3.38	<0.001
Direct	Rejection ⇒ SPA	2.47	0.51	1.47	3.47	0.24	4.85	<0.001
Total	Rejection ⇒ SPA	2.64	0.52	1.63	3.65	0.26	5.13	<0.001

Note. Confidence intervals computed with the method: Standard (Delta method).
